# Scrutiny of *Mycobacterium tuberculosis* 19 kDa antigen proteoforms provides new insights in the lipoglycoprotein biogenesis paradigm

**DOI:** 10.1038/srep43682

**Published:** 2017-03-08

**Authors:** Julien Parra, Julien Marcoux, Isabelle Poncin, Stéphane Canaan, Jean Louis Herrmann, Jérôme Nigou, Odile Burlet-Schiltz, Michel Rivière

**Affiliations:** 1 Institut de Pharmacologie et de Biologie Structurale, Université de Toulouse, CNRS, UPS, France; 2 Aix Marseille Univ, CNRS, EIPL, IMM FR3479, Marseille, France; 3 UMR1173, INSERM, Université de Versailles St Quentin, 78180, Montigny le Bretonneux, France

## Abstract

Post-translational modifications (PTMs) are essential processes conditioning the biophysical properties and biological activities of the vast majority of mature proteins. However, occurrence of several distinct PTMs on a same protein dramatically increases its molecular diversity. The comprehensive understanding of the functionalities resulting from any particular PTM association requires a highly challenging full structural description of the PTM combinations. Here, we report the in-depth exploration of the natural structural diversity of the *M. tuberculosis* (Mtb) virulence associated 19 kDa lipoglycoprotein antigen (LpqH) using intact protein high-resolution mass spectrometry (HR-MS) coupled to liquid chromatography. Combined top-down and bottom-up HR-MS analyses of the purified Mtb LpqH protein allow, for the first time, to uncover a complex repertoire of about 130 molecular species resulting from the intrinsically heterogeneous combination of lipidation and glycosylation together with some truncations. Direct view on the co-occurring PTMs stoichiometry reveals the presence of functionally distinct LpqH lipidation states and indicates that glycosylation is independent from lipidation. This work allowed the identification of a novel unsuspected phosphorylated form of the unprocessed preprolipoglycoprotein totally absent from the current lipoglycoprotein biogenesis pathway and providing new insights into the biogenesis and functional determinants of the mycobacterial lipoglycoprotein interacting with the host immune PRRs.

Post-translational-modifications (PTMs), which consist in the covalent addition of functional groups or in the alteration of amino acids of the gene edited protein sequence, generate diverse structurally and functionally different proteins referred as proteoforms^1^. These PTMs are known to play an essential role in a wide variety of biological processes. However, obtaining a full and accurate structural description of protein PTMs remains most often a considerable challenge that hinders the comprehensive understanding of their biological impacts.

In archaea and bacteria, protein’s PTMs are widely recognized as potential means for pathogens to disturb and evade host defense responses allowing them to survive and to replicate within the infected host. In this context, we recently showed that O-mannosylation is essential for the virulence of the human pathogen *M. tuberculosis* (Mtb), highlighting the determinant role of the secreted manno-proteins in the pathogenicity of Koch’s bacillus^2^,^3^. However, many of these extracellular antigenic glycoproteins are known to or are suspected of also being lipoproteins^4^,^5^. This is notably the case for the 19 kDa secreted lipoglycoprotein LpqH: a major cell wall antigen of Mtb addressed to the Sec protein export pathway by a 21 amino acid secretion signal peptide^6^. *lpqh* homologues are present in many mycobacterial genomes^7^. However, to date, none of the related gene products have been reported to exhibit the Mtb LpqH unique properties that contribute to the pathogenicity of Mtb and of the associated *Mycobacterium tuberculosis* Complex (MTC) mycobacteria. Indeed, Mtb LpqH has profound pleiotropic modulatory effects on the anti-microbial immune response of the infected host, promoting the activation of neutrophils^8^ and CD4+ T cells^9^ or the differentiation of dendritic cells^10^. It is also known to alter the expression and antigen presentation by the macrophage class II major histo-compatibility complex (MHC-II)^11^ by blocking interferon-γ dependent chromatin remodeling at the promoter locus of the MHC-II transcription activator (MHC2TA)^12^ via TLR2 and MAPK signaling. In addition, LpqH is thought to favor Mtb immune evasion and dissemination by inducing TLR2 dependent macrophage apoptosis^13^ while activating TLR2 mediated autophagy^14^, or by acting as an adhesin, promoting phagocytosis through the lectin receptors MMR^15^ or DC-SIGN^16^ on the immune cell surface. Recently, LpqH has been identified as a major component of the membrane vesicles actively secreted by Mtb within infected macrophages^17^ and that contribute to mycobacterial virulence^18^. It is worth mentioning that most of the immunomodulatory effects of LpqH have been ascribed to either the TLR2 agonist activity of the lipoglycoprotein lipidic moiety^9^,^10^,^11^,^12^,^13^,^14^,^18^ or to the presence of mannose motifs binding the immune cell lectins^15^,^16^. However, despite the essential contribution of both the glycosylation and the lipidation in the LpqH’s biological functions^7^,^19^, the detailed structural description of these modifications and in particular the number of hexoses, the precise glycosylation site, the carbohydrate motifs attached to each glycosylation site, or the acylation profile, remain scarce and rough, being mainly inferred from a puzzle of complementary partial structural analyses^20^, lectin binding assays^21^ and genetic evidence^6^. To date, indeed, the complexity of the proteoform repertoire generated by the combined heterogeneity of glycosylation and acylation still strongly hampers the in-depth description of the structural diversity of bacterial lipoglycoproteins and in particular of the Mtb LpqH antigen. However, the accurate structural characterization of modified surface-exposed or secreted microbial proteins is crucial to the comprehensive understanding of how these molecules contribute to the pathogen virulence and evasion of the host’s defense system.

For many years, mass spectrometry (MS) has proven to be the method of choice for the characterization of protein PTMs, and great efforts are pursued to improve PTM identifications either after tryptic digestion^22^ or on intact proteins (top-down) in denaturing^23^ or native^24^ conditions. However, despite the recent impressive in-depth MS-characterization of the Mtb proteome absolute composition and dynamics^25^, the PTM associated structural diversity of the Mtb proteome still remains under-explored^26^.

Up to now, structural features of the mycobacterial lipo and/or glycoproteins have been mostly inferred from tandem mass spectrometry analysis of modified peptides issued from proteolytic digestion of either purified proteins^27^,^28^,^29^,^30^ or complex secretomes^2^,^31^. While these bottom-up approaches are determinant to evidence and characterize the mycobacterial lipo-glycoprotein PTMs, they only provide partial and theoretical combinatorial views of the parent proteins that do not allow reconstructing unambiguously the genuine proteoform repertoires. In parallel, intact protein MS analysis has proven very early its usefulness in resolving the glycoform repertoire of the first described Mtb Apa mannoprotein^32^. However, the huge mass dispersion and overlap resulting from the combination of both heterogeneous glycosylation and lipidation modifications found in lipo-glycoproteins such as LpqH, has remained long time beyond the mass resolution capabilities of conventional mass spectrometers. Recently though, the outstanding performances offered by high resolution mass spectrometry (HR-MS) for the understanding of the micro-heterogeneity of intact proteins^33^,^34^,^35^ together with the development of efficient front-end separation approaches have open promising perspectives for top-down proteomics characterization of mycobacterial proteins post translational modifications^36^,^37^. Indeed, the pioneering top-down MS exploration by Ge *et al*.^34^ of a Mtb secretome readily revealed the presence of glycosylated proteins though these remained unidentified and the glycosylations unlocalized. More recently, CZE-MS analysis of intact proteins secreted by *M. marinum*^35^ enabled the detection of hundreds of different molecular weights, 58 of which were assigned to proteoforms issued from the combination of N-terminal acetylation, signal peptide maturation and methionine excision. Furthermore, these secretome proteomic studies also identified some lipoproteins but without any experimental evidence concerning the nature or localization of the acylation.

In order to gain insights into the LpqH structure-activities relationships, we took advantage of these up-to-date mass spectrometry developments to uncover the long time unapproachable structural molecular diversity associated to the pleiotropic biological activities of the Mtb LpqH. For this purpose, we produced a fully modified recombinant poly-histidine tagged Mtb protein in the *M. smegmatis* heterologous expression system commonly used to express the biologically active Mtb LpqH^15^,^16^,^38^. Indeed *M. smegmatis* possesses all the machinery required to mannosylate and lipidate heterologous Mtb lipoglycoproteins^5^,^6^,^19^,^21^,^27^,^32^,^39^,^40^ but does not express any equivalent endogenous LpqH protein^41^,^42^,^43^ (despite the presence in its genome of the two *lpqh* homologue genes MSMEG_6315 and MSMEG_6316^7^). The Mtb recombinant LpqH protein was then purified independently of its modifications, by affinity purification, for mass spectrometry analyses. Nano-LC-ESI HR-MS analyses of the intact protein identified more than 130 different proteoforms present in the affinity-purified fraction revealing for the first time the full structural complexity of the Mtb LpqH. In addition, this study provides a direct view of the stoichiometry of the different co-occurring PTMs on each proteoforms disclosing the formal limits of the theoretical perimeter of the proteoform repertoire. Finally, the intact protein MS analyses reveal a novel phosphorylated proteoform of the preprolipoprotein that would never have been detected by conventional bottom up strategies based on a-priori searches. This new phosphorylated LpqH molecular species is absent from the current models of the bacterial lipoglycoprotein biosynthesis pathways, and thus, questions about the influence of this modification on the downstream glycosylation-lipidation of the mature LpqH.

## Results

### Intact protein mass spectrometry reveals the complex proteoform pattern of LpqH

**Figure 1:**
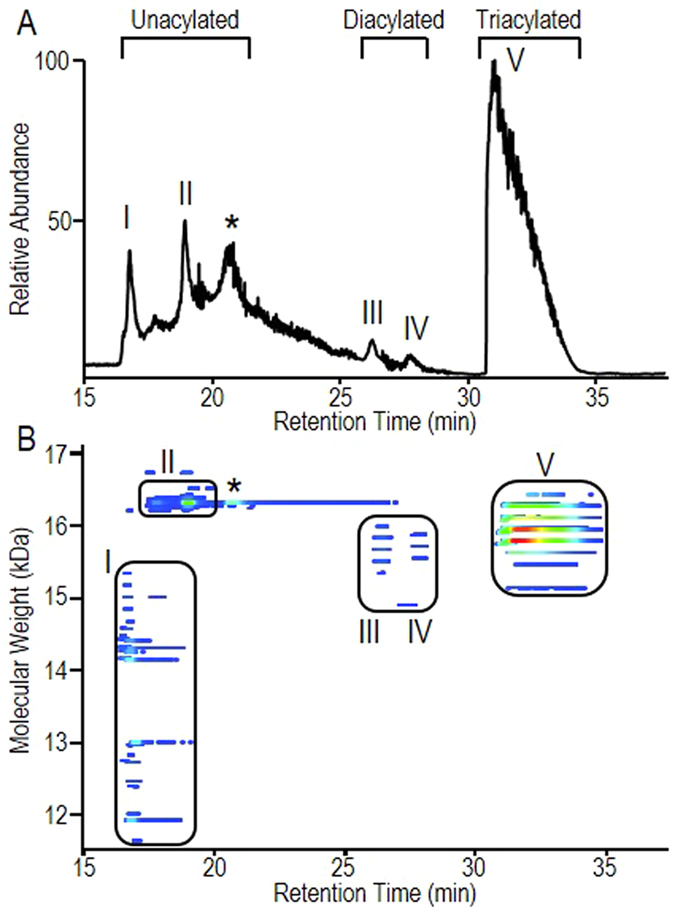
Protein footprint of rLpqH^His^. (**A**) LC-MS separation of the different acylated forms of the Mtb rLpqH^His^ expressed in *M. smegmatis*. The peaks I, II, III/IV and V of the Total Ion Chromatogram correspond to the non-acylated, di-acylated and tri-acylated form of rLpqH^His^, respectively. The corresponding full MS spectra are shown in Fig. S1. (**B**) Proteoform footprint of the LC-MS run generated with RoWinPro^55^, showing the deconvoluted MWs obtained along the gradient. The relative intensities are color-coded (from blue to red). The peak noted by a star corresponds to a co-purified protein of similar MW identified as the *M. smegmatis* his-rich Lumazine Synthase (A0R6M2) (MW 16325 Da).

The combination of hydrophilic glycosylation and hydrophobic lipidation generates a continuum of amphiphilic molecular species rendering the conventional purification strategies complex and weakly adapted to cover the large solubility spectrum of Mtb LpqH^21^. With the aim then of recovering the broadest range of molecular species, histidine tagged recombinant Mtb LpqH was cloned in an *M. smegmatis* expression system^21^ and purified from the cell protein extract by nickel-affinity chromatography. Purification control by coomassie blue stained SDS-PAGE shows a homogeneous fraction appearing as a broad band that was readily attributed to purified recombinant His-tagged LpqH (rLpqH^His^) after western-blotting with anti-His-tag antibodies (Fig. S1A). However, analysis of this affinity-purified protein fraction by LC-MS using a C4 reversed phase chromatography nanocolumn reveals the presence of different peaks, successively noted I, II, *, III, IV, and V (Fig. 1A) that are clearly distinguishable by their averaged mass spectrum (Fig. S1B to G). The 3D proteic footprint (Fig. 1B) generated by deconvolution of the intact protein mass spectra over the full chromatographic window further highlights the molecular complexity of purified LpqH (theoretical MW, 16255 Da) revealing up to 129 different LpqH proteoforms (Table S1) characterized on the basis of their respective mass and chromatographic retention time. Interestingly, three out of the four molecular clusters circled in Fig. 1B contain a series of co-eluted proteoforms differing by constant mass increments of 162 Da. This mass corresponds to that of hexoses and these increments are thus consistent with the expected mannosylation of mycobacterial LpqH. In view then of achieving a comprehensive understanding of the molecular basis for Mtb LpqH’s biological activities, we thoroughly explored the structural molecular diversity of purified rLpqH^Hi^s.

### Truncated forms of LpqH are not lipidated

**Figure 2:**
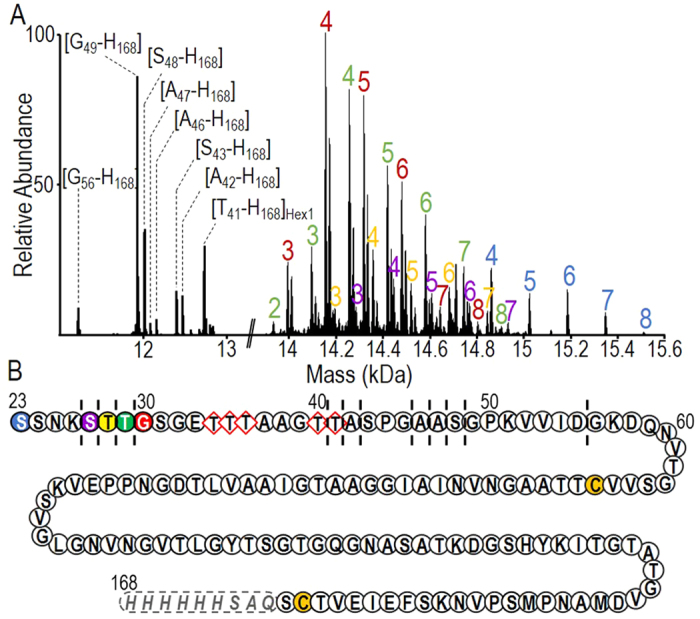
Truncated proteoforms locate up to 8 mannose residues within stretch Gly_30_-Thr_41_. (**A**) Deconvoluted MS spectrum of the chromatographic peak I attributed to non-lipidated N-terminally truncated LpqH species showing the co-elution of low molecular weight unglycosylated forms (peptidic sequence range from [Gly_56_-His_168_ to Thr_41_-His_168_] annotated into brackets) with longer glycosylated forms starting from each color coded N-terminal amino acids figured in the sequence scheme (the colored numbers correspond to the glycosylation degrees of each color coded peptidic sequence). (**B**) Sequence scheme of the non acylated species of LpqH figuring by dashed lines and colored amino acids respectively the N-terminal residue of the different unglycosylated and glycosylated species detected in the peak I; (Amino acid numbering corresponds to that of the full length (gene) sequence and red diamonds indicate the previously proposed LpqH glycosylation sites^6^).

The first chromatographic peak (peak I, Fig. 1A) arises from two groups of molecular species distinguishable by their respective mass ranges (11–13 kDa and 14–15.5 kDa). Mass spectrometry (Fig. 2A) identified the lower molecular mass components as proteoforms of LpqH truncated in their N-terminal region and ranging from Thr_41_ ([T_41_-H_168_], MW = 12735 Da) to Gly_56_ ([G_56_-H_168_], MW = 11221 Da). Interestingly, apart from [T_41_-H_168_], these short proteoforms are likely devoid of any PTM suggesting that both glycosylation and acylation occur on the N-terminal part of the protein sequence, upstream of Ala_42_ (Fig. 2B). The mass spectrum of the higher mass proteoforms at 14–15.5 kDa (Fig. 2A) shows a complex mixture of several glycoforms of five co-eluting longer protein sequences [S_23_/S_27_/T_28_/T_29_/G_30_-H_168_] harboring each up to eight hexose residues. Interestingly, both the longest and the shortest glycosylated proteoforms (respectively [S_23_-H_168_] and [G_30_-H_168_]) accommodate eight hexoses, therefore suggesting that none of the five Ser or Thr residues between these two positions is glycosylated (Fig. 2B). Altogether, the MS data for these co-eluted truncated proteoforms indicate that LpqH is glycosylated on its first N-terminal half on the stretch S_31_-T_41_ that has six potential glycosylation sites, namely one Ser and five Thr residues. These results are in agreement with a previous targeted mutagenesis study^6^ that showed that the concomitant replacement of the five Threonine in between position 33 and 41 by Alanine residues abrogates the binding of LpqH to the lectin Con canavalin A. Moreover, the absence of lipidation for any of these truncated forms is in keeping with the fact that all these molecular species start downstream from the putative acylated residue Cys_22_, part of the lipobox signal shared by bacterial N-terminal lipidated proteins^5^.

### LpqH’s repertoire contains a limited number of diacylated proteoforms

**Figure 3:**
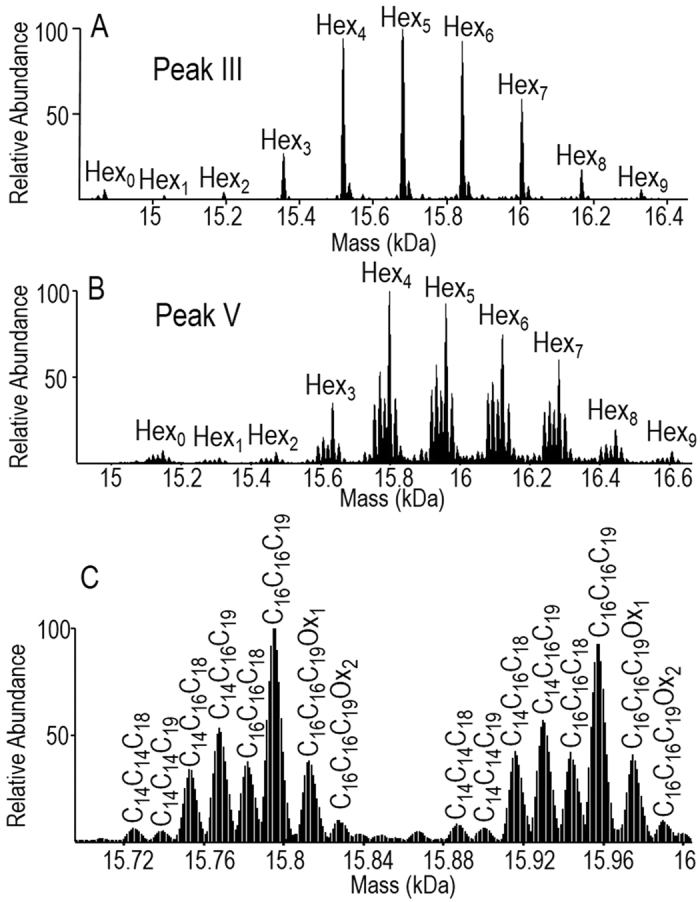
Deconvoluted MS spectra of the lipidated species of rLpqH^His^. (**A**) Glycoforms of the di-palmitoyl-glycerol (GroC_16_C_16_) lipidated rLpqH^His^, (**B**) Deconvoluted MS spectrum of rLpqH^His^ triacylated species showing the molecular diversity generated by the combination of glycosylation and acylation heterogeneity. (**C**) Close up view on the tetra and penta glycosylated species showing the additional heterogeneity brought by the different acylation motifs (Ox: Cysteine oxidation).

The MS analysis of the second cluster consisting of chromatographic peaks III and IV (Fig. 1A) yielded very similar mass spectra; both peaks showing the presence of up to nine molecular ions distant of 162 mass units and that are thereby readily attributable to glycosylated species differing by their glycosylation degrees (Figs 3A and S2). The calculated molecular masses and the observed retention times are consistent with the identification of diacylated LpqH proteoforms bearing a di-palmitoyl-glycerol (GroC_16_C_16_, peak III) and a tuberculostearoyl-palmitoyl-glycerol (GroC_16_C_19_, peak IV) moiety on the N-terminal Cys_22_. This assignment is further supported by the observed baseline separation according to their relative hydrophobicity, as these two molecular species differ by three methylene (CH_2_) units. LpqH diacyl-forms have only been observed in the case of an *M. bovis* BCG Lnt mutant invalidated for N-acyltransferase^20^. This observation thus raises the question of the putative origins of these compounds and further studies are in course to try to determine whether the diacylated LpqH proteoforms detected here correspond to LpqH biogenesis intermediates or result from catabolic degradation of the final triacylated forms.

### Triacylated LpqH shows a very high diversity of proteoforms

The MS analysis of the last eluted proteoforms from the broad and intense peak (peak V, Fig. 1A) consistently identified these most hydrophobic species as triacylated glycoforms of LpqH, evidencing for the first time the simultaneous glycosylation and triacylation of a protein. The deconvoluted mass spectrum acquired over peak V (Fig. 3B and C), fully captures the complexity arising from the combination of the two heterogeneous PTMs, with the dispersion resulting from the number of substituting carbohydrates overlapping with the heterogeneity of the lipidic moiety. In total, 71 different LpqH proteoforms were detected by MS in peak V (Table S1). These result from different combinations of the 10 glycosylation states (Fig. 3B) and the 6 possible fatty acid combinations (Fig. 3C) substituting the *sn1, sn2* and amino terminal positions of the N-terminal S-glyceryl-cysteine residue. A closer look at the ion distribution within each glycoform clearly shows four preferential acyl-forms corresponding to the most probable fatty acid combinations (C_14_C_16_C_18_), (C_14_C_16_C_19_), (C_16_C_16_C_18_), and (C_16_C_16_C_19_). Moreover, quantification of the MS signal intensities shows a predominance of the (C_16_C_16_C_19_) arrangement accounting for about 70% of the total triacylated LpqH proteoforms (Fig. S3).

### Top-down MS analysis identifies LpqH’s acylation site

**Figure 4:**
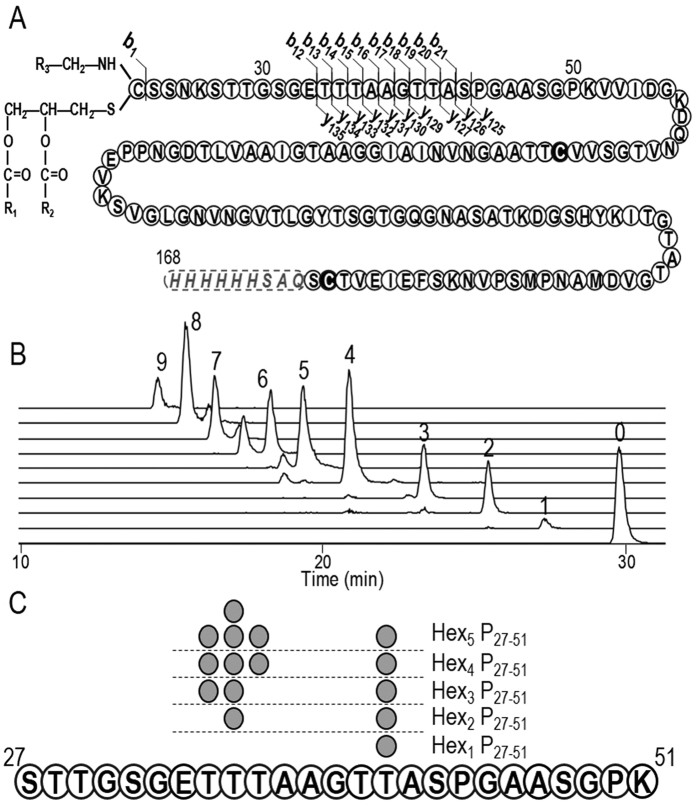
Mass spectrometry analysis of the rLpqH^His^ microheterogeneity. (**A**) Fragmentation pattern of the hepta glycosyl triacylated rLpqH^His^ proteoform [Hex_7_,GroC_16_C_16_C_19_] obtained by top-down CID (Gro: Glycerol; R_1_ + R_2_ + R_3_ = C_51_; Precursor ion: 1810.02^9+^). (**B**) Chromatographic separation of the different glycoforms (from 0 to 9 hexoses) of the tryptic peptide [S_27_-K_51_]. (**C**) Localization of the first 4 sequential glycosylation sites on rLpqH^His^ obtained from bottom-up analysis of the [S_27_-K_51_] glycoforms.

We then addressed the micro-heterogeneity (the precise arrangement and localization of the PTMs) of these proteoforms by tandem MS fragmentation of the intact proteins (top-down MS). The collision induced dissociation (CID) fragmentation spectra of the major LpqH glyco-acyl-forms all reveal a main characteristic fragment ion resulting from the neutral loss of every single glycan motif from the intact precursors (Fig. S4). Although it confirms the predicted glycosylation degree (GD) deduced from the molecular mass of the intact precursor, this ion reveals little about the localization of the glycosylation site. Further activation of the intact protein precursor yielded series of both *y* and *b* fragment ions. The substantial mass excess of the *b* series ions, and in particular of the *b*_*1*_ ion (at m/z = 934.79 in Fig. S4A), is clearly consistent with an N-terminal N-acyl, S-diacylglycerol cysteine tri-acylated by two palmitic (C_16_) and one tuberculostearic (C_19_) fatty acids for the proteoform of MW = 16282 Da. Besides, it is worth mentioning that most of the identified y and b fragment ions arise from the same limited protein stretch concentrating all of the putative glycosylation sites (Fig. 4A)^6^. However, although these fully ‘deglycosylated’ fragment ions suggest that the preferential fragmentation pathway of the intact protein is glycan driven; they do not provide any information on the glycosylation sites or glycan structures. Complementary attempts to fragment the intact LpqH glyco-acyl-forms by electron transfer dissociation (ETD, known to preserve labile glycosylation motifs) were unsuccessful. No dissociation could be observed despite efficient electron transfer (ETnoD)^44^, irrespective of the charge state or glycosylation degree of the precursor (Fig. S5).

### Bottom-up ETD-MS^2^ analysis reveals the glycosylation microheterogeneity of LpqH

Bottom-up ETD LC-MS^2^ analysis of trypsin digested LpqH clearly identifies S_27_-K_51_ as the only glycosylated peptide, bearing from zero to nine hexoses (Figs S6 and 4B). This identification is confirmed by the consistent separation of the different glycoforms of the peptide on the C18 reverse chromatography nanocolumn according to their expected relative polarity inferred from their respective GDs (Fig. 4B). Thorough analysis of the ETD MS^2^ spectra of the peptide glycoforms with up to five hexoses (Fig. S6) shows unambiguous series of *c* and *z* fragment ions that readily reveal the glycosylation sites. This analysis shows that each glycosylated peptide is a unique positional isomer, which suggests that the glycosylation process is sequential (Fig. 4C). The glycosylation sites could not however be determined reliably for the glycoforms with higher GDs (with six to nine hexoses). Nevertheless, our data definitively confirm four out of the five glycosylation sites previously suggested^6^, namely Thr_34_, Thr_35_, Thr_36_, and Thr_41_. In addition, analysis of the glycoforms with GD ≤ 5 reveals, that glycosylation is not randomly distributed on the potential glycosylation sites but concern progressively the different Thr in the following order: Thr_41_, Thr_35_, Thr_34_, and then Thr_36_. It is worth mentioning that we previously reported a similar preferential progression of the glycosylation process in the case of the Fasciclin containing protein of *M. smegmatis*^2^. It can be reasonably suspected that such determinism may be due most probably to a different accessibility of the different glycosylation sites for the protein mannosyl transferase (PMT). Finally, the fact that no dihexosyl motifs were detected for the glycopeptides with GD < 5 suggests that the four potential glycosylation sites have to be substituted first by PMT before the glycan chain is elongated further by PimE mannosyl transferase^2^. These observations provide valuable clues for further investigations of the molecular mechanisms underlying the mannosylation of bacterial protein.

### Intact protein MS reveals a novel phosphorylated form of LpqH

**Figure 5:**
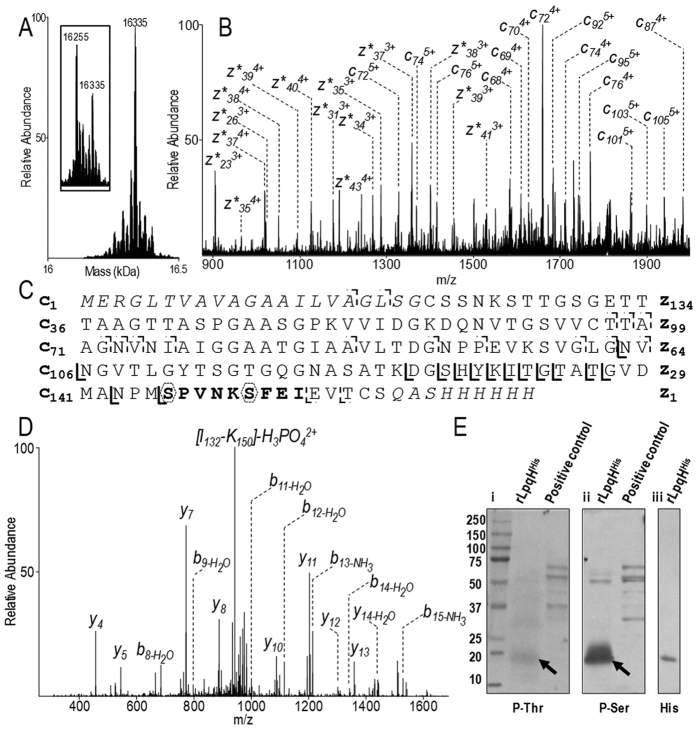
Evidence of phosphorylation of the LpqH preprolipoprotein. (**A**) Deconvoluted mass spectra of chromatographic peak II, showing a full-length phosphorylated rLpqH^His^ proteoform (16335 Da). The inset shows the loss of the phosphate group (mass shift of −80 Da) after treatment with alkaline phosphatase. (**B**) Top-down ETD fragmentation localizes the phosphorylation site on the C-terminal side of the protein. Phosphorylated *z* fragments are marked with a star. (**C**) rLpqH^His^ sequence showing the *z* and *c* fragments generated by ETD: the phosphorylated fragments ions are indicated by bold lines while dashed line noted fragmentation sites correspond to non-modified ions. Dashed polygons indicate potential phosphosites. (**D**) Bottom-up CID fragmentation of peptide [I_132_-K_150_] evidencing the neutral loss of 98 Da corresponding to the dissociation of a phosphoric acid. (**E**) rLpqH^His^ (arrows) western blot detection with i) anti-phospho-threonine (clone 1E11), ii) anti-phospho-serine (clone 1C8) and iii) anti-His-Tag antibodies, supporting the presence of a phosphorylated Serine on rLpqH^His^. Positive controls are phosphoproteins mixture provided by antibodies manufacturers.

A detailed examination of the full repertoire of proteoforms detected by intact protein MS reveals that peak II (Fig. 1A) corresponds to an unexpected polar species of high molecular mass (16335 Da, Fig. 5A). This proteoform, apparently devoid of any of the PTMs discussed above, is 80 Da heavier than the full-length preprolipoprotein (theoretical mass, 16255 Da), suggesting that it is phosphorylated. This interpretation is supported by the analysis of the complementary *c* and *z* fragment ions observed in the top-down ETD fragmentation spectrum (Fig. 5B) of the intact protein precursor ion (m/z = 1090.01^15+^). Indeed, the series of *c* ions, in particular the *c*_*17*_ and *c*_*19*_ ions (dashed line Fig. 5C), clearly evidence a full size LpqH proteoform with an unmodified N-terminal export signaling sequence. Furthermore, the series of *z* ions upstream of *z*_*23*_ all present an 80 Da mass excess suggesting that the modification is localized on the C-terminal stretch S_146_-I_154_ (Fig. 5C). To confirm further that this proteoform corresponds to a phosphorylated form of the preprolipoprotein, the purified LpqH was submitted to dephosphorylation by bovine alkaline phosphatase. After two hours, mass spectrometry analysis of the treated fraction shows a peak at MW = 16255 Da (Insert Fig. 5A) corresponding to the mass expected for the preprolipoprotein. This downsizing of 80 Da of the molecular mass of the major component, consecutively to the dephosphorylation treatment, is consistent with the loss of a phosphate group and thereby definitively supports the phosphorylation of the preprolipoprotein. Further MS analyses of the trypsin digest of the affinity-purified LpqH clearly evidences a precursor ion whose observed mass is 80 Da in excess of that expected for the tryptic peptide I_132_-K_150_ (observed mass, 1982.90 Da; theoretical mass, 1902.91 Da). Phosphorylation of this peptide is readily supported by its CID-fragmentation spectrum that shows a characteristic intense fragment ion at m/z = 943.64 (MW, 1885.28 Da), consistent with the neutral loss of a labile phosphoric acid group (H_3_PO_4_; MW, 97.99 Da) from the parent ion (Fig. 5D). The converging results from both approaches independently support the presence of protein phosphorylation on the C-terminal part of full-length LpqH. Moreover, the fact that tyrosine is absent from any of the two partially overlapping peptides I_132_-K_150_ and S_146_-I_154_ suggests that this modification is a phosphorylation of one of these peptides’ Thr or Ser residues, most probably Ser_146_, which is part of both. To assess this assumption, we probed affinity-purified LpqH by Western blot using a panel of anti phospho-serine and anti-phospho-threonine antibodies. The intense band observed with the monoclonal anti-phosphoserine antibodies 1C8 (Fig. 5E) and 4A9 (Fig. S7) clearly highlights the presence of a phosphorylated serine. The weak but detectable signal obtained with the anti P-Thr antibodies 1E11 (Fig. 5E) or 14B3 (Fig. S7) suggest furthermore that a threonine may also be phosphorylated. These data thus confirm the MS results that the polar high molecular mass LpqH proteoform is an unexpected phosphorylated preprolipoprotein.

## Discussion

**Figure 6:**
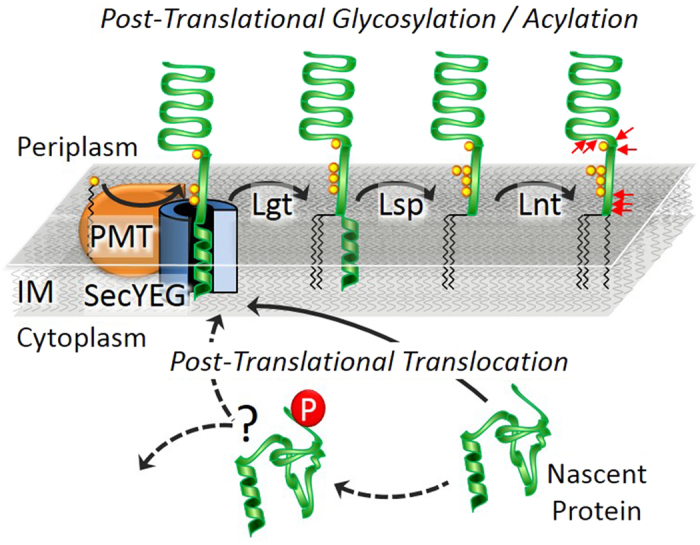
General scheme localizing the different proteoforms of LpqH into an integrated model of the lipoglycoprotein biogenesis pathway^47^,^51^. The unfolded LpqH pre-prolipoprotein, addressed via its N-terminal signal peptide (SP), to the SecYEG machinery is translocated across the inner membrane to reach the periplasmic compartment where it undergoes maturation through successive mannosylation (by the PMT and PimE) and lipidation and SP excision (by the Lgt/Lsp/Lnt enzyme triad). The absence of both glycosylation and lipidation on the phosphorylated preprolipoprotein suggests that phosphorylation occurs prior to periplasmic maturation. The early putative position of this peculiar phosphorylated proteoform in the biogenesis scheme, interrogates about its roles and its possible outcome on the lipoglycoprotein biosynthesis. (The SecA/B chaperonin protein complex has been omitted for clarity; the red arrows symbolize the N terminal extremities of the truncated forms of rLpqH^His^ identified herein).

We report here the thorough characterization of the proteoform repertoire of recombinant LpqH, a lipoglycoprotein antigen from Mtb, using a combination of up to date top-down (intact protein) and bottom-up mass spectrometry. Unexpectedly, about 130 proteoforms—in their vast majority differently acylated, glycosylated and truncated forms of the mature protein—were identified with isotopic resolution. From a biological point of view, besides the strictly analytical relevance of this work, the accurate MS characterization of the PTM combinations harbored by the proteoforms of intact LpqH provides valuable clues on the intriguing subject of the possible interdependency of acylation and glycosylation. Indeed, just like Gram-negative lipoproteins, mycobacterial lipoglycoproteins are generally considered to be first translated in the cytoplasm into a pre-mature unfolded precursor containing an N-terminal signal peptide (SP) that addresses the preprolipoprotein to the Sec secretory system (Fig. 6)^45^,^46^. The SP is then cleaved after membrane translocation in the periplasm, where the protein undergoes lipidation through three successive modifications. Besides, according to Vanderven *et al*.^47^, the initiation of glycosylation by PMT is concomitant to protein translocation in the periplasm. Then both processes are temporally associated with the protein’s translocation across the membrane via the Sec translocon. According to this, our observations are consistent with a sequential model where the glycosylation of the translocated preproliporotein occurs before it is released in the inner membrane (IM) to undergo the lipidation-associated modifications that produce the fully matured triacylated lipoprotein (Fig. 6). However, the low but detectable presence of lipidated proteoforms devoid of glycosylation together with the constant distribution observed for the acyl forms within the glycoform series strongly suggest that glycosylation is not a prerequisite for lipidation and that the two processes may occur independently. However, we cannot exclude that these populations are observable only because of the dynamic imbalance of a substrate-saturated system caused by the overexpression of the protein in *M. smegmatis*.

The in-depth mass spectrometry characterizations of the nature, the number, the localization and the stoichiometry of the PTMs defining each proteoforms of LpqH afford also significant insights into their respective putative functionalities. As for example, the precise determination of both the degree and sites of glycosylation of antigenic bacterial proteins is essential for the molecular understanding of their biological significance. Indeed, these hydrophilic decorations are suspected of determining the proteolytic processing of the soluble forms of mannolipoprotein antigens^6^, but have also been shown to contribute directly to host-pathogen interactions by helping the pathogen colonize its target cell^3^ or evade the host immune system^48^. However, the present work is the first straight chemical determination of the glycosylation of LpqH since Garbe *et al*.^21^ initially reported the evidence of LpqH glycosylation by lectin binding, more than twenty years ago. Here, combined MS analyses provide a complete and detailed structural characterization of LpqH’s glycosylation profile, namely up to nine hexoses distributed over four glycosylation sites located on the N-terminal portion of the protein. Moreover, the fact that all the truncated forms of LpqH identified here arise also from the same portion of the protein strongly support a causality relationship between glycosylation and proteolysis as previously presumed^6^. On the other hand, until recently, bacterial lipoproteins were believed to exist in only one specific lipid-modified structure and the triacylated form was largely claimed to be the only form naturally present in mycobacteria^27^,^45^,^49^. However, the two di-acylated forms of LpqH separated and characterized in this study strongly contradict this assumption. It may be argued that these forms are artifacts resulting from uncontrolled degradation in the recombinant protein expression system. However, we cannot totally exclude the possibility that they occur naturally through incomplete maturation^20^ or catabolic lipolysis processes regulated by the growth environment as shown for *S. Aureus*^45^. Bearing in mind the ability of immune system PRRs to discriminate between the di- and tri-acylated lipoprotein, respectively via the TLR2/6 and TLR2/1 heterodimers, one can reasonably suspect that, whatever their origin, these di-acylated forms of LpqH (although less abundant than the tri-acylated forms) may contribute to the fine tuning of the immune cell’s response to the pathogen. Further reliable structural and quantitative descriptions of the different acylation states will be required to shed light on the structure functions relationships involved in N-terminal lipidation and the immune response to LpqH. Interestingly, the unique available structure of LpqH (Fig. S8) only encompasses residues 49–159. Given the lack of structural information on the highly modified N-terminal region, we think that our detailed results are of upmost interest, showing a huge molecular heterogeneity in the first 41 residues and probably explaining why the N-terminal part has not been crystallized so far. The foremost breakthrough of this study is the characterization by intact protein MS of a phosphorylated proteoform of the preprolipoprotein that has remained undetected so far by bottom-up wide proteomic or targeted phosphoproteomic approaches using conventional peptide database searches. This unexpected finding demonstrates the pertinence of the HR-MS analysis of intact proteins for the characterization of proteoforms. These results also raise new biological questions, in particular about the meaning of this non-mature phosphorylated proteoform that interrogates the current paradigm for lipoprotein biogenesis in mycobacteria. Indeed, it is widely considered that Mtb protein phosphorylation occurs in the cytoplasmic compartment^50^ in contrast to the extracellular protein glycosylation and lipidation processes (discussed above). Thus, a reasonable conclusion drawn from these current models is that phosphorylation of the preprolipoprotein most probably occurs in the cytoplasm before translocation. However, the absence of any glycosylation or lipidation concomitant to the phosphorylation is intriguing and suggests that this modification is exclusive. Therefore, keeping in mind that phosphorylation is a very general regulation signal for protein activity; it is tempting to speculate that the early phosphorylation of the preprolipoprotein may influence the processing of LpqH associated with membrane translocation. From a structural point of view, the phosphorylated peptide I_132_-K_150_ encompasses the last flexible loop and beta strands 9 and 10. The sidechains of the four putative phosphosites contained in this peptide are all accessible to the solvent. However, Ser146, which seems to be the main phosphosite, as suggested by our western blot and MS data, sits on a flexible loop more favorable to the phosphorylating kinase access than the three threonine residues located on the constrained beta strand 9 (Fig. S8). Nevertheless, based on the partial available structure it is not possible to predict the impact of the phosphorylation on any conformational rearrangement of C-terminal region of the protein. Then in order to gain insights into the effect of phosphorylation on the LpqH modifications, it will be essential to determine whether the phosphorylated preprolipoprotein is a transient form that requires dephosphorylation to be further glycosylated and lipidated; or an alternate form that addresses the protein to a different pathway that remains to be identified. These different hypotheses raise many questions, i.e.: which mycobacterial kinase is involved? which precise step of the secretion pathway is affected by the phosphorylation? what is the outcome of the phosphorylated form? and does phosphorylation contribute to the quality control of recombinant lipoglycoproteins? Although additional work will be required to understand the biological significance of this finding, this unexpected phosphorylated form of LpqH’s preprolipoprotein adds a novel piece to the biogenesis scheme of lipoglycoproteins and may provide some unprecedented clues about a phosphorylation dependent regulation of the mycobacterial lipoglycoproteins biogenesis.

In conclusion and to our knowledge, this detailed description of the complex structural diversity of a dually post-translationally modified protein, is the first direct overview of the molecular distribution and stoichiometry of the different co-appearing modifications of an amphiphilic lipoglycoprotein at the level of the proteoform. It is worth mentioning that the molecular diversity observed here for LpqH could not have been dissected so thoroughly using only a conventional proteolysis assisted “bottom-up” approach. Indeed, in this case the identification of the protein is inferred from the characterization of the constituting peptides but the link between the modified peptide and the proteoform from which it originates is totally wiped out. Here we overcome this limitation by using, for the first time, intact protein high resolution mass spectrometry^33^,^34^,^35^,^44^ to gain access into the natural diversity of a mycobacterial lipoglycoprotein. This unprecedented work allows us detailing the structure of the diverse molecular species that are present, in a so-called purified fraction of LpqH, and that represent effective components susceptible to contribute differently to the reported biological activities of LpqH. Moreover, our results open the way for further comprehensive analyses of the relative proportion of these different proteoforms and of the potential factors that could affect these proportions. Such exploration will probably help for a better understanding of the putative roles of the PTMs harbored by the respective LpqH molecular species in the alteration of the host immune response. Finally, the wider use of this approach for the analysis of other virulence associated mycobacterial lipoglycoproteins^51^ (such as LprG^52^ or LppX^53^, both presumed to complex and to transport major antigenic glycolipids and lipoglycans) would be of major interest to further decipher the roles and the structure-functions relationships of the respective PTMs in the function of these proteins.

## Materials and Methods

### Bacterial strains and growth conditions

All cloning experiments were performed in *E. coli* DH10B cells (Invitrogen) and cells were grown at 37 °C in Luria-Bertani broth (LB) or on agar plates and supplemented with 200 μg/mL hygromycin B (Euromedex). The *M. smegmatis* strain mc2155 GroEL1ΔC^54^ used for expression experiments was grown at 37 °C under stirring (220 rpm) in Middlebrook 7H9 broth (Difco) supplemented with 0.05% Tween80 (v/v), 0.2% Glycerol (v/v), 0.5% BSA (w/v), 0.2% Glucose and 150 mM NaCl or on Middlebrook 7H11 agar. Hygromycin B at 50 μg/mL was used for the selection of transformed bacteria.

### Preparation of cell extract and protein purification

LpqH gene, encoding 19-kDa protein with its predicted signal sequence, was amplified by PCR (Forward primer 5′AAATACC-ATGGAGCGTGGACTGACGGTCG3′ (NcoI), Reverse primer 5′AACATGAAGCTTGGGAACAGGTCACCTCGATTTCG3′ (HindIII)) from *M. tuberculosis* genomic DNA was gel purified, digested by NcoI and HindIII restriction enzymes and cloned after ligation into pMyC expression vector. The LpqH gene was cloned in frame with the C-terminal Histidine tag contained into the pMyC plasmid. pMyC-LpqH was transformed into the recombinant *M. smegmatis* mc2155 GroEL1ΔC strain. Two liters of both recombinant strains were grown in Middlebrook 7H9 complete medium for 3 days at 37 °C under shaking. Expression of rLpqH^His^ was induced for 16 h by adding acetamide (Sigma-Aldrich) to a final concentration of 0.2%. Cells were then harvested at 4 °C by centrifugation for 10 min at 3 000 × g and the cell pellets were resuspended in 30 mL of ice-cold buffer A (10 mM Tris/HCl pH 8.0 containing 150 mM NaCl and 1% N-Lauroylsarcosine). Cells were then passed three times through a French press set at 1 100 psi to ensure complete cell lysis and further sonicated twice (15-s bursts at 90 W with 15-s ice cooling periods between bursts) using a Branson sonifier 450 in ice. Samples were then centrifuged for 30 min at 15 000 × g and the supernatant was loaded onto a ProBond Ni2+-agarose column (Invitrogen) (5 mg of recombinant protein per mL of resin) preequilibrated with buffer A. After loading samples, the column was washed with 5 column volumes (CV) of buffer A followed by 5 CV of buffer A containing 25 mM of imidazole. Recombinant protein was then eluted at 100 mM and 250 mM of imidazole. The eluted fractions were analysed by performing SDS/PAGE on Any kD gel from Biorad. Fractions containing rLpqH^His^ were pooled, concentrated to 1 mg/ml and load onto a gel filtration S200. Eluted and pure recombinant LpqH were pooled concentrated to 1 mg/ml and stored at −80 °C. Due to the absence of tryptophan, the protein concentration was evaluated using the extinction molar coefficient ε280 = 0.183 and correlated with the quantification performed on SDS-PAGE.

### Western Blot Analysis

2 μg of recombinant purified rLpqH^His^ was reduced in 1X Laemmli buffer for 5 min at 95 °C. After electrophoresis (12% acrylamide SDS-PAGE), proteins were transferred to a nitrocellulose membrane (TransBlot^®^-TurboTM, Bio-Rad). The membrane was blocked with 1% Bovine Serum Albumin (Sigma-Aldrich) in TBS buffer and incubated over night with the PhosphoDetect™ anti-phosphoserine (1C8, 4A3, 4A9, 16B4 Merck Millipore), anti-phosphothreonine (1E11, 4D11, and 14B3, Merck Millipore), and anti-His (Sigma-Aldrich) antibodies at 4 °C. After washing four times with TBST-buffer (TBS with 0.1% Tween20), the membrane was incubated at room temperature for 1 h with anti-mouse horseradish peroxidase-conjugated secondary antibody (Santa Cruz Biotechnology) diluted in TBST-buffer. The detected protein signals were visualized by an enhanced chemiluminescence reaction system using the Amersham Biosciences ECL kit and revealed with the ChemiDoc™ Touch Imaging System (BioRad).

### In-gel protein digestion

20 μg of recombinant purified LpqH^His^ was reduced and alkylated by successive incubation in 25 mM DTT for 5 min at 95 °C and 100 mM chloroacetamide for 30 min at room temperature in the dark. After separation of proteins by 12% acrylamide SDS-PAGE and Coomassie Blue staining, the band corresponding to the rLpqH^His^ was cut and washed at least 3 times in 25 mM ammonium bicarbonate, acetonitrile (1:1) for 15 min at 37 °C. Proteins were digested by incubating each gel slice with 400 ng of modified sequencing grade trypsin (Promega) in 50 mM ammonium bicarbonate overnight at 37 °C. The resulting peptides were extracted from the gel using two successive incubations in 10% formic acid, acetonitrile (1:1) for 15 min at 37 °C. The two collected extractions were pooled, dried in a Speed-Vac, and resuspended with 20 μL of 2% acetonitrile, 0.05% TFA for the nanoLC-MS^2^ analysis.

### Enzymatic protein dephosphorylation

20 μg of recombinant purified LpqH^His^ was dephosphorylated with 31 units of bovin alkaline phosphatase (Sigma-Aldrich) in 5 mM Tris pH 7.9, 10 mM NaCl, 1 mM MgCl_2_ and 0.1 mM DTT for 2 h at 30 °C. At this time, 5 μL of the reaction was diluted directly in the loading buffer (5% acetonitrile, 0.05%TFA) prior to injection in LC-MS.

### Top down LC-MS and LC-MS^2^

NanoLC-MS and MS^2^ analyses of intact rLpqH^His^ were performed on a nanoRS UHPLC system (Dionex) coupled to an ETD-enabled LTQ-Orbitrap Velos mass spectrometer (Thermo Fisher Scientific), with fluoranthene as reagent anion. 5 μL of sample was loaded on a C4-precolumn (300 μm ID × 5 mm, Thermo Fisher Scientific) at 20 μL/min in 2% acetonitrile, 0.05% TFA. After 5 min desalting, the precolumn was switched online with the analytical C4 nanocolumn (75 μm ID × 15 cm, in-house packed with C4 Reprosil) equilibrated in 95% solvent A (0.2% formic acid) and 5% solvent B (0.2% formic acid in acetonitrile). Proteins were eluted using the following gradient of solvent B at 300 nL/min flow rate: 5 to 40% during 5 min; 40 to 100% during 33 min. The LTQ-Orbitrap Velos was operated either in single MS or in data-dependent acquisition mode with the XCalibur software. MS scans were acquired in the 500–2000 m/z range with the resolution set to a value of 60,000. For top-down experiments, survey scan MS were acquired in the same way, with a resolution at 30,000. Precursor ions were selected from an inclusion list established thanks to previous MS analyses and were fragmented in CID or ETD and the resulting fragment ions were analyzed in the Orbitrap, at a resolution of 60,000. Isolation width was set at 5 m/z, NCE was set at a value of 35% for CID fragmentation, and activation time was fixed to 20 ms for ETD.

### Bottom-up LC-MS^2^

Trypsin digests were analyzed by nanoLC-MS^2^ using a nanoRS UHPLC system (Dionex) coupled to an ETD-enabled LTQ-Orbitrap Velos mass spectrometer (Thermo Fisher Scientific), with fluoranthene as reagent anion. 5 μL of sample was loaded on a C18-precolumn (300 μm ID × 5 mm, Thermo Fisher Scientific) at 20 μL/min in 2% acetonitrile, 0.05% TFA. After 5 min desalting, the precolumn was switched online with the analytical C18 nanocolumn (75 μm ID × 15 cm, in-house packed with C18 Reprosil) equilibrated in 95% solvent A (0.2% formic acid) and 5% solvent B (80% acetonitrile, 0.2% formic acid). Peptides were eluted using the following gradient of solvent B at 300 nL/min flow rate: 5 to 25% gradient during 75 min; 25 to 50% during 30 min; 50 to 100% during 10 min. The LTQ-Orbitrap Velos was operated in data-dependent acquisition mode with the XCalibur software. Survey scan MS were acquired in the Orbitrap on the 300–2000 m/z range with the resolution set to a value of 60,000. The 20 most intense ions per survey scan were selected for ETD fragmentation and the resulting fragments were analyzed in the linear trap (LTQ). Activation time was dependent on the precursor charge state, and supplemental activation was enabled.

Both top-down and bottom-up data have been deposited to the MassIVE repository with the dataset identifier MSV000080396 (ftp://MSV000080396@massive.ucsd.edu).

## Additional Information

**How to cite this article:** Parra, J. *et al*. Scrutiny of *Mycobacterium tuberculosis* 19 kDa antigen proteoforms provides new insights in the lipoglycoprotein biogenesis paradigm. *Sci. Rep.* **7**, 43682; doi: 10.1038/srep43682 (2017).

**Publisher's note:** Springer Nature remains neutral with regard to jurisdictional claims in published maps and institutional affiliations.

## Supplementary Material

Supplementary Information
