# Supplementary material for: Scrutiny of Mycobacterium tuberculosis 19 kDa antigen proteoforms provides new insights in the lipoglycoprotein biogenesis paradigm
Source: Sci Rep. 2017 Mar 8;7:43682. doi: 10.1038/srep43682 (PMC5341126; doi:10.1038/srep43682)
Supplement: Supplementary Information [file srep43682-s1.pdf]

Supplementary information

Scrutiny of *Mycobacterium tuberculosis* 19 kDa antigen proteoforms provides new insights in the lipoglycoprotein biogenesis paradigm

Julien Parra[1,+], Julien Marcoux[1,+], Isabelle Poncin[2], Stéphane Canaan[2], Jean Louis Herrmann[3], Jérôme Nigou[1], Odile Burlet-Schiltz[1,*], Michel Rivière[1,*]

[1]Institut de Pharmacologie et de Biologie Structurale, Université de Toulouse, CNRS, UPS, France

[2]CNRS – Aix-Marseille Université, Enzymologie Interfaciale et Physiologie de la Lipolyse UMR7282, Marseille, France

[3]UMR1173, INSERM, Université de Versailles St Quentin, 78180, Montigny le Bretonneux, France

*Correspondence should be addressed to M.R. (Michel.riviere@ipbs.fr) or O.B-S. (email: Odile.schiltz@ipbs.fr)

[+]these authors contributed equally to this work

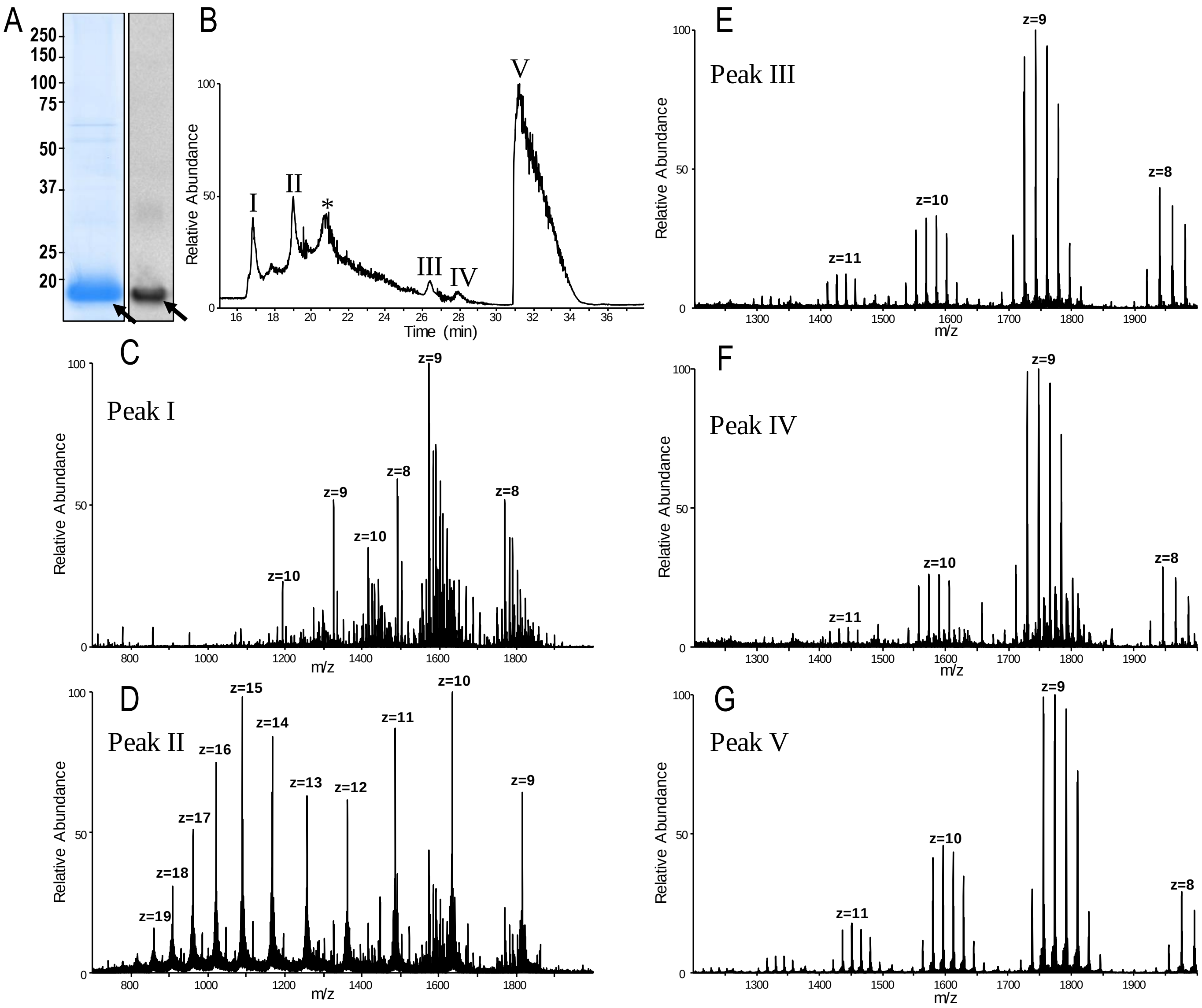

Figure S1. A single band showing different chromatographic peaks upon LC-MS analysis. (**A**) SDS-PAGE and anti His-tag Western Blotting of the recombinant LpqH^{His} (arrows). (**B** to **F**). Averaged full MS scans of the chromatographic peaks I, II, III, IV and V attributed to the different classes of LpqH proteoforms observed by the LC-MS analysis. These spectra show charge state distributions ranging from 8+ to 19+.

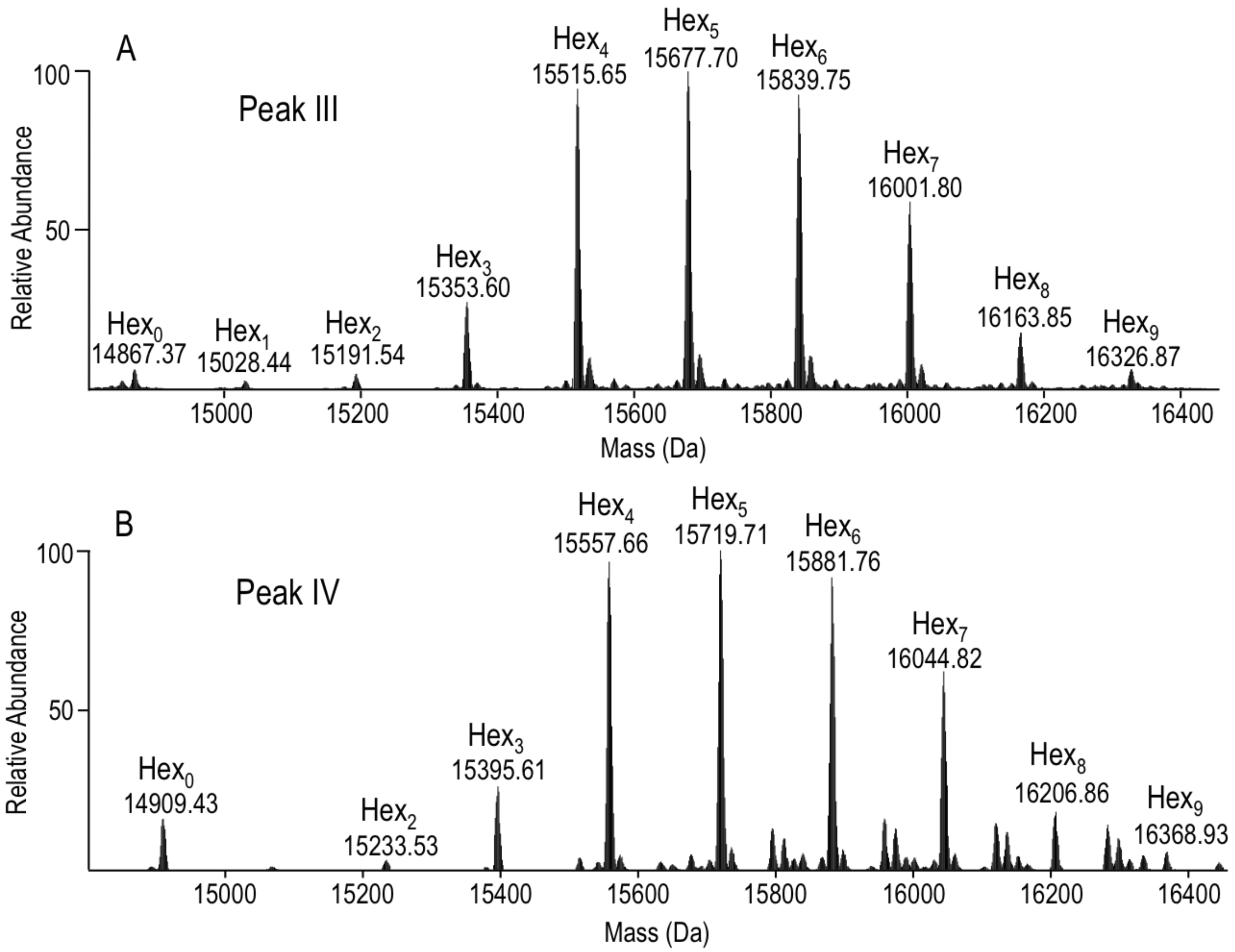

Figure S2. Deconvoluted MS spectra of the diacylated species of rLpqH^{His}. Different glycoforms of rLpqH^{His} [C_{22}-H_{168}] acylated with (**A**) a di-palmitoyl-glycerol ($GroC_{16}C_{16}$), or (**B**) a tuberculostearoyl-palmitoyl-glycerol ($GroC_{16}C_{19}$).

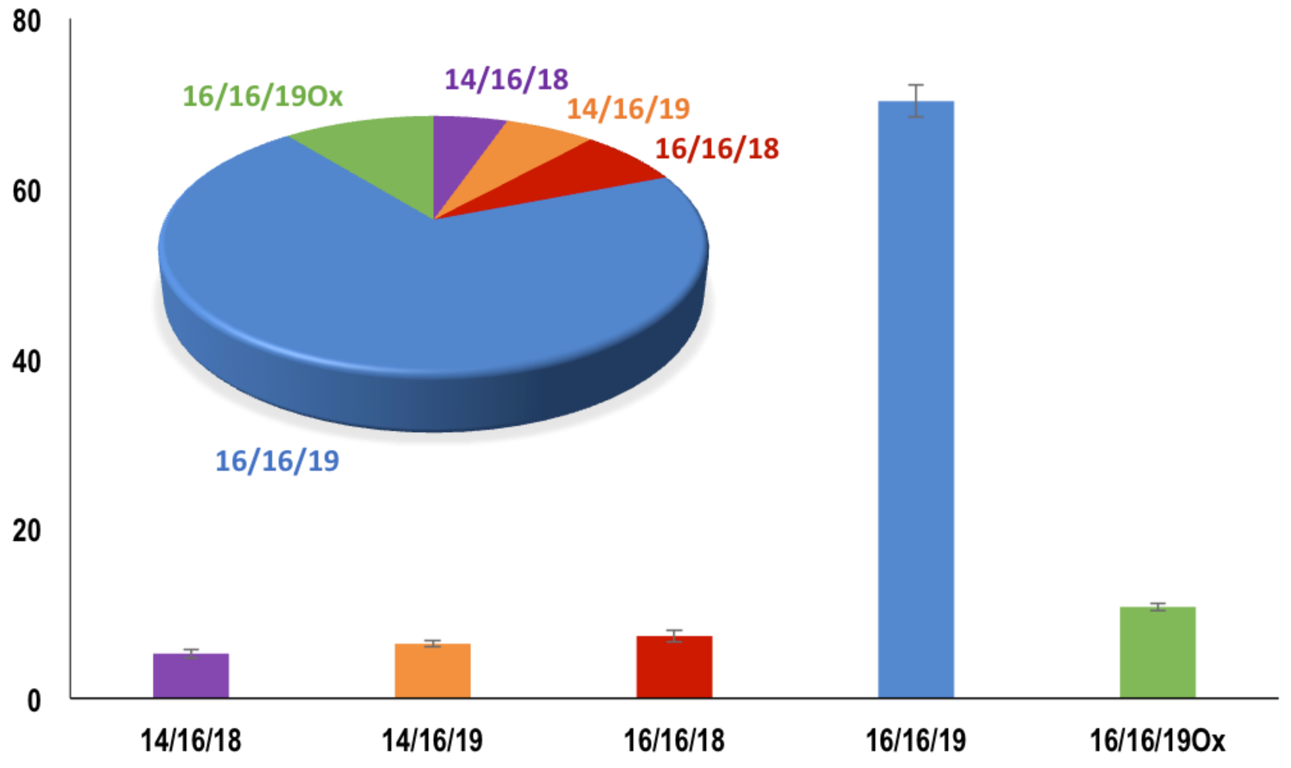

Figure S3. Relative abundance of each lipidic composition harbored by the tri-acylated proteoforms of rLpqH^{His}. The error bars represent the standard deviation from the mean of 5 deconvoluted glycoforms.

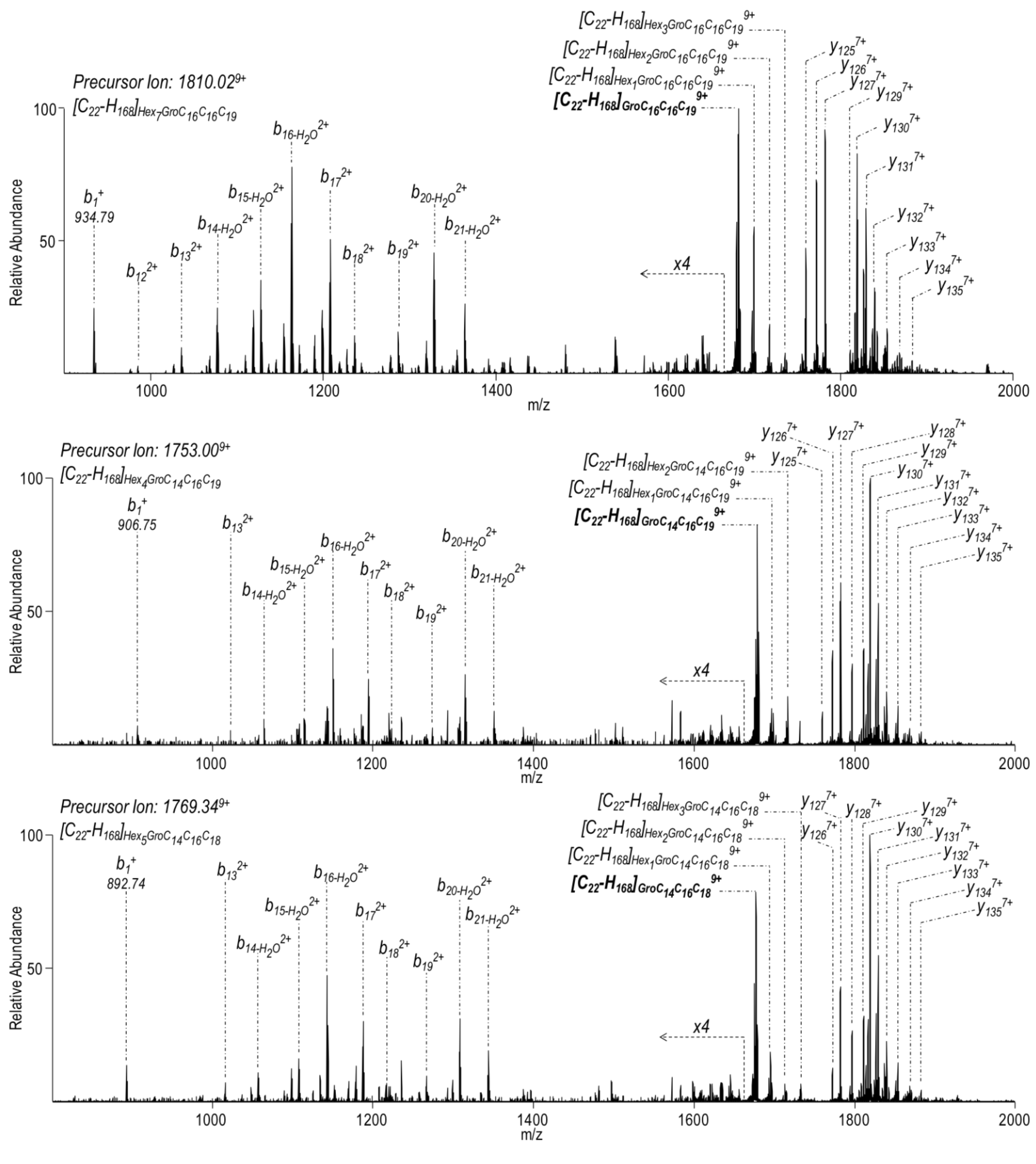

Figure S4. Top-down CID fragmentation of triacylated rLpqH^{His}. (**A**) $[C_{22}\text{-}H_{168}]Hex_7GroC_{16}C_{16}C_{19}$ (Precursor ion: 1810.02^{9+}), (**B**) $[C_{22}\text{-}H_{168}]Hex_4GroC_{14}C_{16}C_{19}$ (Precursor ion: 1753.00^{9+}) and (**C**) $[C_{22}\text{-}H_{168}]Hex_5GroC_{14}C_{16}C_{18}$ (Precursor ion: 1769.34^{9+}).

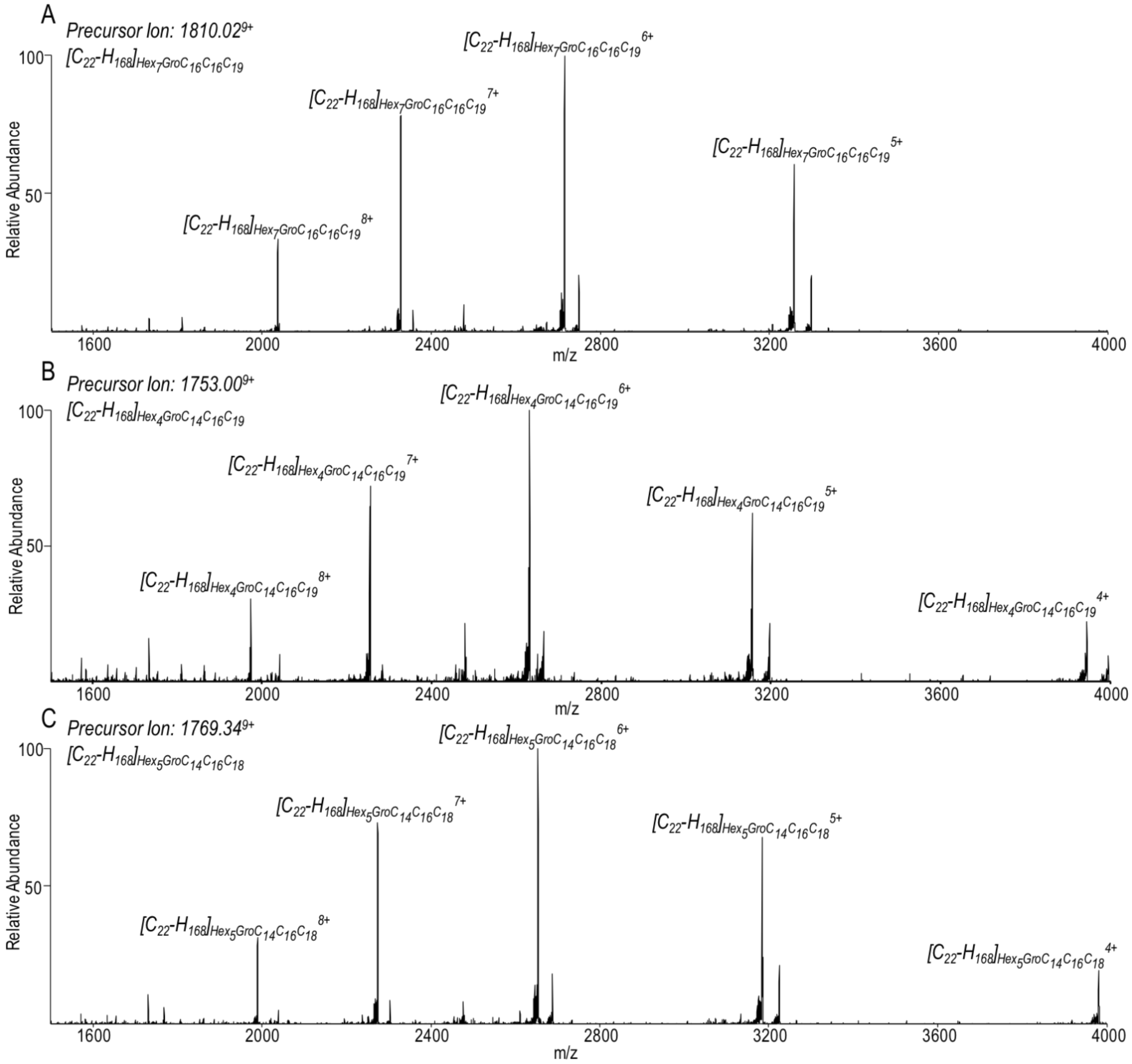

Figure S5. Top-down ETnoD spectra of the triacylated rLpqH^{His}. (**A**) $[C_{22}$-$H_{168}]Hex_7GroC_{16}C_{16}C_{19}$ (Precursor ion: 1810.02^{9+}), (**B**) $[C_{22}$-$H_{168}]Hex_4GroC_{14}C_{16}C_{19}$ (Precursor ion: 1753.00^{9+}) and (**C**) $[C_{22}$-$H_{168}]Hex_5GroC_{14}C_{16}C_{18}$ (Precursor ion: 1769.34^{9+}).

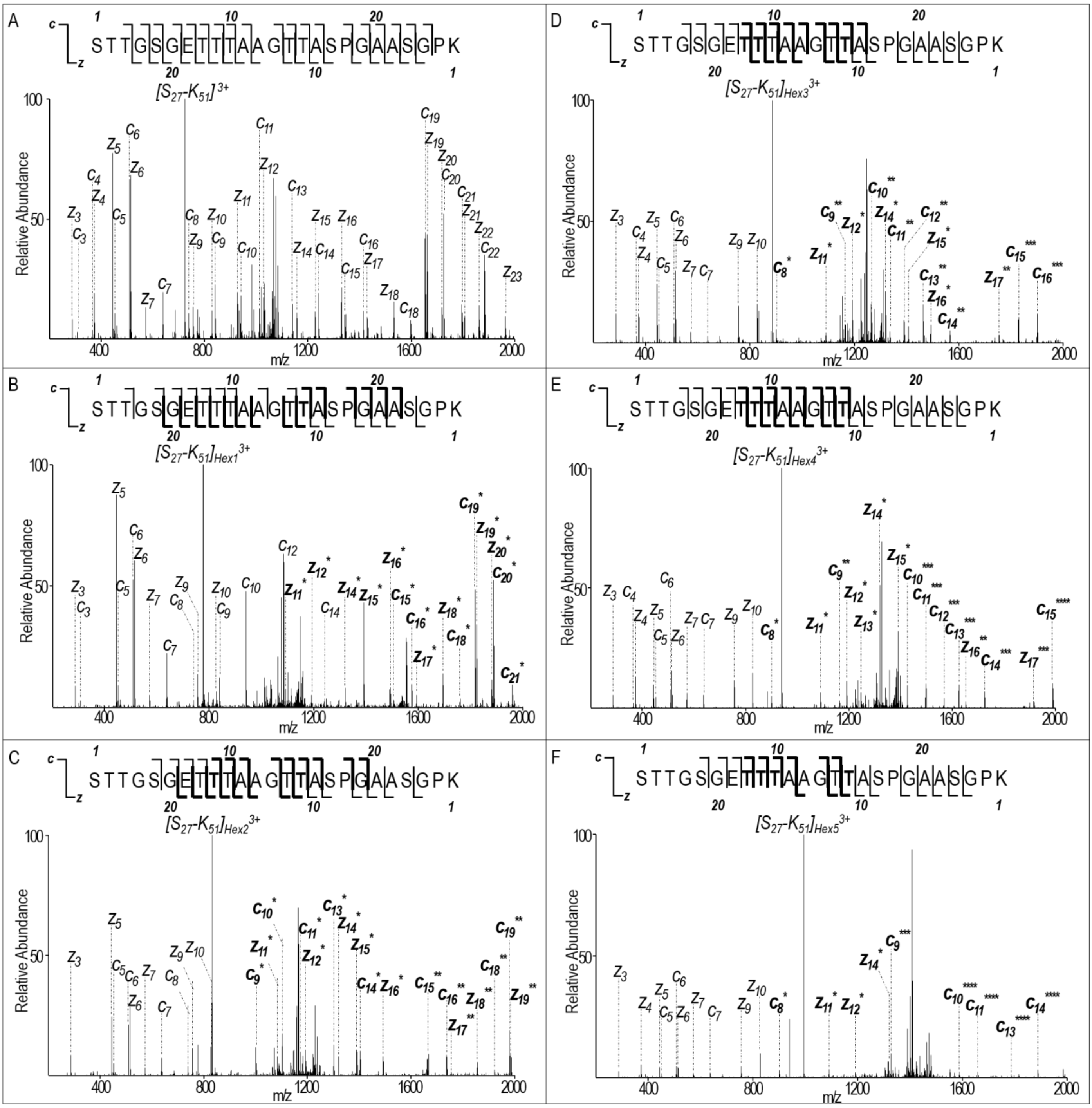

Figure S6. Bottom-up ETD fragmentation of tryptic peptide S_{27}-K_{51}. (**A**) non glycosylated (Precursor ion: 723.013+), (**B**) mono-glycosylated (Precursor ion: 777.033+), (**C**) di-glycosylated (Precursor ion: 831.043+), (**D**) tri-glycosylated (Precursor ion: 885.063+), (**E**) tetra-glycosylated (Precursor ion: 939.083+) and (**F**) penta-glycosylated (Precursor ion: 993.103+). Glycosylation sites and fragments are marked in bold, and number of glycosylation units are indicated by stars on spectra.

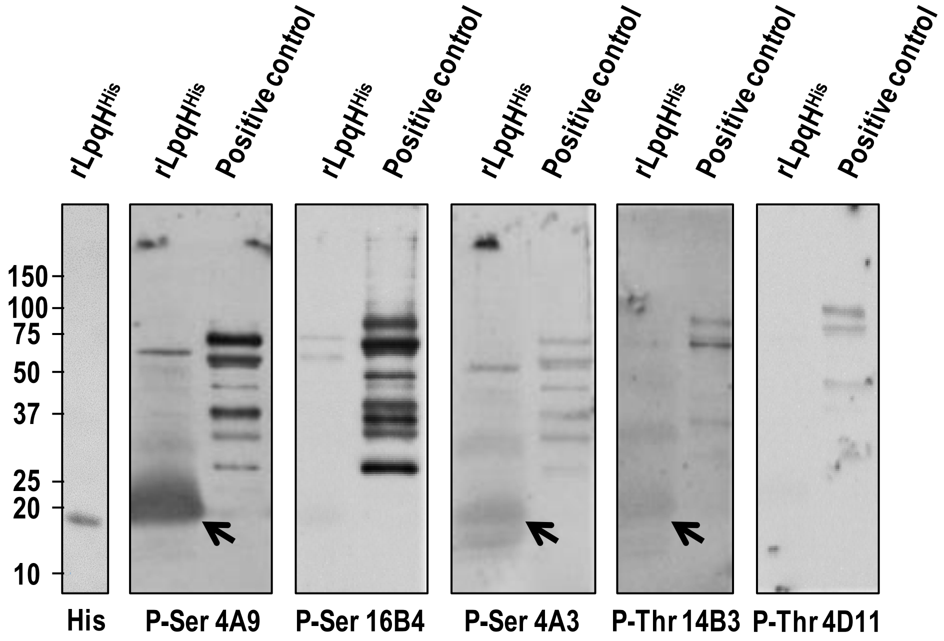

Figure S7. Western blotting of rLpqH^{His} with Anti-Phospho-Serine and Anti-Phospho-Threonine antibodies. Positive controls are phosphoproteins mixture provided by antibodies manufacturers.

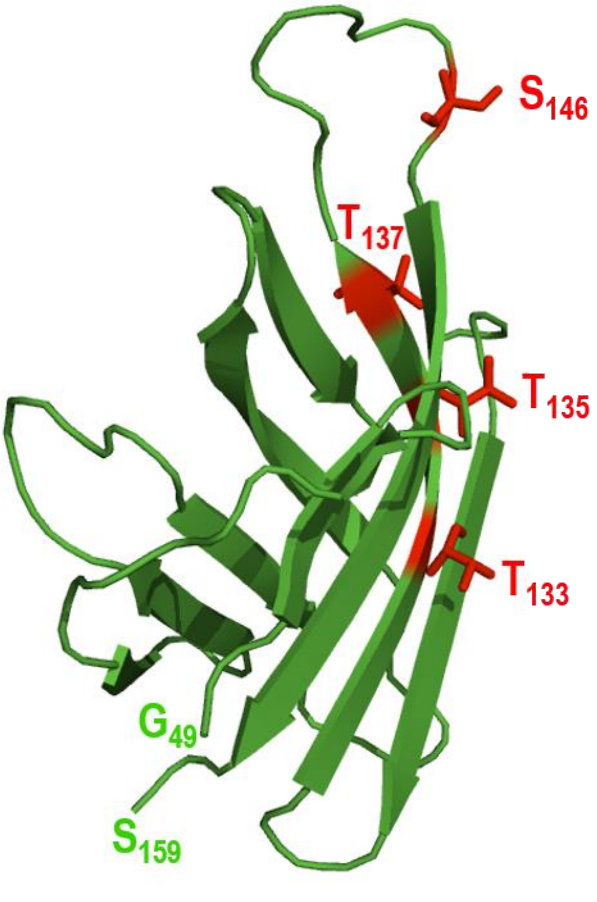

Figure S8. Structural model of LpqH (PDB 4ZJM), and localization of the four potential phosphorylation sites (indicated in red). Only the fragment [G_{49}-S_{159}] has been resolved in this model, excluding the N-terminal part of the protein encompassing all of the glycosylation and acylation sites.

Table S1. List of proteoforms of $rLpqH^{His}$ identified. ΔM tolerance was set to 2.5 Da.

Chromatographic peak	Experimental Mass (Da)	Theoretical Mass (Da)	ΔM (Da)	Sequence	PTMs
I	11 221.44	11 222.19	0.75	$[G_{56}-H_{168}]$	
I	11 648.69	11 648.70	0.01	$[V_{52}-H_{168}]$	
I	11 930.87	11 931.03	0.16	$[G_{49}-H_{168}]$	
I	12 017.90	12 018.11	0.21	$[S_{48}-H_{168}]$	
I	12 088.94	12 089.19	0.25	$[A_{47}-H_{168}]$	
I	12 158.97	12 160.27	1.30	$[A_{46}-H_{168}]$	
I	12 401.09	12 401.51	0.42	$[S_{43}-H_{168}]$	
I	12 472.12	12 472.59	0.47	$[A_{42}-H_{168}]$	
I	12 735.23	12 735.74	0.51	$[T_{41}-H_{168}]$	Hex_1
I	13 933.69	13 932.79	0.90	$[T_{29}-H_{168}]$	Hex_2
I	13 993.75	13 993.74	0.01	$[G_{30}-H_{168}]$	Hex_3
I	14 095.78	14 094.84	0.94	$[T_{29}-H_{168}]$	Hex_3
I	14 155.79	14 155.79	0.00	$[G_{30}-H_{168}]$	Hex_4
I	14 195.82	14 195.94	0.12	$[T_{28}-H_{168}]$	Hex_3
I	14 256.84	14 256.89	0.05	$[T_{29}-H_{168}]$	Hex_4
I	14 283.81	14 283.04	0.77	$[S_{27}-H_{168}]$	Hex_3

Chromatographic peak	Experimental Mass (Da)	Theoretical Mass (Da)	ΔM (Da)	Sequence	PTMs
**I**	14 317.84	14 317.84	0.00	[G_{30}-H_{168}]	Hex_5
**I**	14 357.88	14 357.99	0.11	[T_{28}-H_{168}]	Hex_4
**I**	14 418.89	14 418.94	0.05	[T_{29}-H_{168}]	Hex_5
**I**	14 444.89	14 445.09	0.20	[S_{27}-H_{168}]	Hex_4
**I**	14 479.90	14 479.89	0.01	[G_{30}-H_{168}]	Hex_6
**I**	14 519.94	14 520.04	0.10	[T_{28}-H_{168}]	Hex_5
**I**	14 580.94	14 580.99	0.05	[T_{29}-H_{168}]	Hex_6
**I**	14 607.00	14 607.14	0.14	[S_{27}-H_{168}]	Hex_5
**I**	14 641.96	14 641.94	0.02	[G_{30}-H_{168}]	Hex_7
**I**	14 682.00	14 682.09	0.09	[T_{28}-H_{168}]	Hex_6
**I**	14 699.13	14 699.50	0.37	[S_{23}-H_{168}]	Hex_3
**I**	14 743.00	14 743.04	0.04	[T_{29}-H_{168}]	Hex_7
**I**	14 769.04	14 769.19	0.15	[S_{27}-H_{168}]	Hex_6
**I**	14 804.02	14 803.99	0.03	[G_{30}-H_{168}]	Hex_8
**I**	14 844.04	14 844.14	0.10	[T_{28}-H_{168}]	Hex_7
**I**	14 861.09	14 861.55	0.46	[S_{23}-H_{168}]	Hex_4
**I**	14 905.05	14 905.09	0.04	[T_{29}-H_{168}]	Hex_8

**Chromatographic peak**	**Experimental Mass (Da)**	**Theoretical Mass (Da)**	**ΔM (Da)**	**Sequence**	**PTMs**
**I**	14 932.11	14 931.24	0.87	[S_{23}-H_{168}]	Hex_7
**I**	15 024.18	15 023.60	0.58	[S_{23}-H_{168}]	Hex_5
**I**	15 186.23	15 185.65	0.58	[S_{23}-H_{168}]	Hex_6
**I**	15 348.35	15 347.70	0.65	[S_{23}-H_{168}]	Hex_7
**I**	15 510.34	15 509.75	0.59	[S_{23}-H_{168}]	Hex_8
**II**	16 335.01	16 334.74	0.27	[M_1-H_{168}]	HPO_3
**III**	14 867.37	14 867.35	0.02	[C_{22}-H_{168}]	$GroC_{16}C_{16}$
**III**	15 028.44	15 029.40	0.96	[C_{22}-H_{168}]	$Hex_1GroC_{16}C_{16}$
**III**	15 191.54	15 191.45	0.09	[C_{22}-H_{168}]	$Hex_2GroC_{16}C_{16}$
**III**	15 353.60	15 353.50	0.10	[C_{22}-H_{168}]	$Hex_3GroC_{16}C_{16}$
**III**	15 515.65	15 515.55	0.10	[C_{22}-H_{168}]	$Hex_4GroC_{16}C_{16}$
**III**	15 677.70	15 677.60	0.10	[C_{22}-H_{168}]	$Hex_5GroC_{16}C_{16}$
**III**	15 839.75	15 839.65	0.10	[C_{22}-H_{168}]	$Hex_6GroC_{16}C_{16}$
**III**	16 001.80	16 001.70	0.10	[C_{22}-H_{168}]	$Hex_7GroC_{16}C_{16}$
**III**	16 163.85	16 163.75	0.10	[C_{22}-H_{168}]	$Hex_8GroC_{16}C_{16}$
**III**	16 326.87	16 325.80	1.07	[C_{22}-H_{168}]	$Hex_9GroC_{16}C_{16}$
**IV**	14 909.43	14 909.43	0.00	[C_{22}-H_{168}]	$GroC_{16}C_{19}$

Chromatographic peak	Experimental Mass (Da)	Theoretical Mass (Da)	ΔM (Da)	Sequence	PTMs
**IV**	15 233.53	15 233.53	0.00	[C_{22}-H_{168}]	$Hex_2GroC_{16}C_{19}$
**IV**	15 395.61	15 395.58	0.03	[C_{22}-H_{168}]	$Hex_3GroC_{16}C_{19}$
**IV**	15 557.66	15 557.63	0.03	[C_{22}-H_{168}]	$Hex_4GroC_{16}C_{19}$
**IV**	15 719.71	15 719.68	0.03	[C_{22}-H_{168}]	$Hex_5GroC_{16}C_{19}$
**IV**	15 881.76	15 881.73	0.03	[C_{22}-H_{168}]	$Hex_6GroC_{16}C_{19}$
**IV**	16 044.82	16 043.78	1.04	[C_{22}-H_{168}]	$Hex_7GroC_{16}C_{19}$
**IV**	16 206.86	16 205.83	1.03	[C_{22}-H_{168}]	$Hex_8GroC_{16}C_{19}$
**IV**	16 368.93	16 367.88	1.05	[C_{22}-H_{168}]	$Hex_9GroC_{16}C_{19}$
**V**	15 106.64	15 105.76	0.88	[C_{22}-H_{168}]	$GroC_{14}C_{16}C_{18}$
**V**	15 117.64	15 119.79	2.15	[C_{22}-H_{168}]	$GroC_{14}C_{16}C_{19}$
**V**	15 131.64	15 133.81	2.17	[C_{22}-H_{168}]	$GroC_{16}C_{16}C_{18}$
**V**	15 146.68	15 147.84	1.16	[C_{22}-H_{168}]	$GroC_{16}C_{16}C_{19}$
**V**	15 163.67	15 163.83	0.16	[C_{22}-H_{168}]	$GroC_{16}C_{16}C_{19}Ox_1$
**V**	15 177.64	15 179.83	2.19	[C_{22}-H_{168}]	$GroC_{16}C_{16}C_{19}Ox_2$
**V**	15 251.67	15 253.79	2.12	[C_{22}-H_{168}]	$Hex_1GroC_{14}C_{14}C_{19}$
**V**	15 267.74	15 267.81	0.07	[C_{22}-H_{168}]	$Hex_1GroC_{14}C_{16}C_{18}$
**V**	15 280.70	15 281.84	1.14	[C_{22}-H_{168}]	$Hex_1GroC_{14}C_{16}C_{19}$

Chromatographic peak	Experimental Mass (Da)	Theoretical Mass (Da)	ΔM (Da)	Sequence	PTMs
**V**	15 294.74	15 295.86	1.12	[C_{22}-H_{168}]	$Hex_1GroC_{16}C_{16}C_{18}$
**V**	15 307.73	15 309.89	2.16	[C_{22}-H_{168}]	$Hex_1GroC_{16}C_{16}C_{19}$
**V**	15 323.72	15 325.88	2.16	[C_{22}-H_{168}]	$Hex_1GroC_{16}C_{16}C_{19}Ox_1$
**V**	15 427.79	15 429.86	2.07	[C_{22}-H_{168}]	$Hex_2GroC_{14}C_{16}C_{18}$
**V**	15 442.80	15 443.89	1.09	[C_{22}-H_{168}]	$Hex_2GroC_{14}C_{16}C_{19}$
**V**	15 455.80	15 457.91	2.11	[C_{22}-H_{168}]	$Hex_2GroC_{16}C_{16}C_{18}$
**V**	15 470.83	15 471.94	1.11	[C_{22}-H_{168}]	$Hex_2GroC_{16}C_{16}C_{19}$
**V**	15 488.83	15 487.93	0.90	[C_{22}-H_{168}]	$Hex_2GroC_{16}C_{16}C_{19}Ox_1$
**V**	15 503.80	15 503.93	0.13	[C_{22}-H_{168}]	$Hex_2GroC_{16}C_{16}C_{19}Ox_2$
**V**	15 576.85	15 577.89	1.04	[C_{22}-H_{168}]	$Hex_3GroC_{14}C_{14}C_{19}$
**V**	15 591.87	15 591.91	0.04	[C_{22}-H_{168}]	$Hex_3GroC_{14}C_{16}C_{18}$
**V**	15 605.88	15 605.94	0.06	[C_{22}-H_{168}]	$Hex_3GroC_{14}C_{16}C_{19}$
**V**	15 618.88	15 619.96	1.08	[C_{22}-H_{168}]	$Hex_3GroC_{16}C_{16}C_{18}$
**V**	15 632.91	15 633.99	1.08	[C_{22}-H_{168}]	$Hex_3GroC_{16}C_{16}C_{19}$
**V**	15 649.90	15 649.98	0.08	[C_{22}-H_{168}]	$Hex_3GroC_{16}C_{16}C_{19}Ox_1$
**V**	15 665.90	15 665.98	0.08	[C_{22}-H_{168}]	$Hex_3GroC_{16}C_{16}C_{19}Ox_2$
**V**	15 725.89	15 725.91	0.02	[C_{22}-H_{168}]	$Hex_4GroC_{14}C_{14}C_{18}$

Chromatographic peak	Experimental Mass (Da)	Theoretical Mass (Da)	ΔM (Da)	Sequence	PTMs
**V**	15 739.91	15 739.94	0.03	[C_{22}-H_{168}]	$Hex_4GroC_{14}C_{14}C_{19}$
**V**	15 753.92	15 753.96	0.04	[C_{22}-H_{168}]	$Hex_4GroC_{14}C_{16}C_{18}$
**V**	15 767.93	15 767.99	0.06	[C_{22}-H_{168}]	$Hex_4GroC_{14}C_{16}C_{19}$
**V**	15 781.94	15 782.01	0.07	[C_{22}-H_{168}]	$Hex_4GroC_{16}C_{16}C_{18}$
**V**	15 795.96	15 796.04	0.08	[C_{22}-H_{168}]	$Hex_4GroC_{16}C_{16}C_{19}$
**V**	15 812.96	15 812.03	0.93	[C_{22}-H_{168}]	$Hex_4GroC_{16}C_{16}C_{19}Ox_1$
**V**	15 827.95	15 828.03	0.08	[C_{22}-H_{168}]	$Hex_4GroC_{16}C_{16}C_{19}Ox_2$
**V**	15 887.94	15 887.96	0.02	[C_{22}-H_{168}]	$Hex_5GroC_{14}C_{14}C_{18}$
**V**	15 900.95	15 901.99	1.04	[C_{22}-H_{168}]	$Hex_5GroC_{14}C_{14}C_{19}$
**V**	15 915.97	15 916.01	0.04	[C_{22}-H_{168}]	$Hex_5GroC_{14}C_{16}C_{18}$
**V**	15 929.98	15 930.04	0.06	[C_{22}-H_{168}]	$Hex_5GroC_{14}C_{16}C_{19}$
**V**	15 943.99	15 944.06	0.07	[C_{22}-H_{168}]	$Hex_5GroC_{16}C_{16}C_{18}$
**V**	15 958.00	15 958.09	0.09	[C_{22}-H_{168}]	$Hex_5GroC_{16}C_{16}C_{19}$
**V**	15 975.00	15 974.08	0.92	[C_{22}-H_{168}]	$Hex_5GroC_{16}C_{16}C_{19}Ox_1$
**V**	15 989.99	15 990.08	0.09	[C_{22}-H_{168}]	$Hex_5GroC_{16}C_{16}C_{19}Ox_2$
**V**	16 049.98	16 050.01	0.03	[C_{22}-H_{168}]	$Hex_6GroC_{14}C_{14}C_{18}$
**V**	16 062.01	16 064.04	2.03	[C_{22}-H_{168}]	$Hex_6GroC_{14}C_{14}C_{19}$

**Chromatographic peak**	**Experimental Mass (Da)**	**Theoretical Mass (Da)**	**ΔM (Da)**	**Sequence**	**PTMs**
**V**	16 078.01	16 078.06	0.05	[C_{22}-H_{168}]	$Hex_6GroC_{14}C_{16}C_{18}$
**V**	16 092.02	16 092.09	0.07	[C_{22}-H_{168}]	$Hex_6GroC_{14}C_{16}C_{19}$
**V**	16 106.03	16 106.11	0.08	[C_{22}-H_{168}]	$Hex_6GroC_{16}C_{16}C_{18}$
**V**	16 120.05	16 120.14	0.09	[C_{22}-H_{168}]	$Hex_6GroC_{16}C_{16}C_{19}$
**V**	16 137.05	16 136.13	0.92	[C_{22}-H_{168}]	$Hex_6GroC_{16}C_{16}C_{19}Ox_1$
**V**	16 153.04	16 152.13	0.91	[C_{22}-H_{168}]	$Hex_6GroC_{16}C_{16}C_{19}Ox_2$
**V**	16 211.05	16 212.06	1.01	[C_{22}-H_{168}]	$Hex_7GroC_{14}C_{14}C_{18}$
**V**	16 226.06	16 226.09	0.03	[C_{22}-H_{168}]	$Hex_7GroC_{14}C_{14}C_{19}$
**V**	16 240.06	16 240.11	0.05	[C_{22}-H_{168}]	$Hex_7GroC_{14}C_{16}C_{18}$
**V**	16 254.07	16 254.14	0.07	[C_{22}-H_{168}]	$Hex_7GroC_{14}C_{16}C_{19}$
**V**	16 268.08	16 268.16	0.08	[C_{22}-H_{168}]	$Hex_7GroC_{16}C_{16}C_{18}$
**V**	16 282.09	16 282.19	0.10	[C_{22}-H_{168}]	$Hex_7GroC_{16}C_{16}C_{19}$
**V**	16 298.10	16 298.18	0.08	[C_{22}-H_{168}]	$Hex_7GroC_{16}C_{16}C_{19}Ox_1$
**V**	16 315.09	16 314.18	0.91	[C_{22}-H_{168}]	$Hex_7GroC_{16}C_{16}C_{19}Ox_2$
**V**	16 375.09	16 374.11	0.98	[C_{22}-H_{168}]	$Hex_8GroC_{14}C_{14}C_{18}$
**V**	16 402.12	16 402.16	0.04	[C_{22}-H_{168}]	$Hex_8GroC_{14}C_{16}C_{18}$
**V**	16 417.13	16 416.19	0.94	[C_{22}-H_{168}]	$Hex_8GroC_{14}C_{16}C_{19}$

Chromatographic peak	Experimental Mass (Da)	Theoretical Mass (Da)	ΔM (Da)	Sequence	PTMs
**V**	16 430.16	16 430.21	0.05	[C_{22}-H_{168}]	$Hex_8GroC_{16}C_{16}C_{18}$
**V**	16 444.14	16 444.24	0.10	[C_{22}-H_{168}]	$Hex_8GroC_{16}C_{16}C_{19}$
**V**	16 461.15	16 460.23	0.92	[C_{22}-H_{168}]	$Hex_8GroC_{16}C_{16}C_{19}Ox_1$
**V**	16 477.15	16 476.23	0.92	[C_{22}-H_{168}]	$Hex_8GroC_{16}C_{16}C_{19}Ox_2$
**V**	16 550.16	16 550.19	0.03	[C_{22}-H_{168}]	$Hex_9GroC_{14}C_{14}C_{19}$
**V**	16 564.13	16 564.21	0.08	[C_{22}-H_{168}]	$Hex_9GroC_{14}C_{16}C_{18}$
**V**	16 578.18	16 578.24	0.06	[C_{22}-H_{168}]	$Hex_9GroC_{14}C_{16}C_{19}$
**V**	16 592.21	16 592.26	0.05	[C_{22}-H_{168}]	$Hex_9GroC_{16}C_{16}C_{18}$
**V**	16 606.21	16 606.29	0.08	[C_{22}-H_{168}]	$Hex_9GroC_{16}C_{16}C_{19}$
**V**	16 623.23	16 622.28	0.95	[C_{22}-H_{168}]	$Hex_9GroC_{16}C_{16}C_{19}Ox_1$
**V**	16 636.20	16 638.28	2.08	[C_{22}-H_{168}]	$Hex_9GroC_{16}C_{16}C_{19}Ox_2$
